# Immunopathogenesis of Juvenile Systemic Sclerosis

**DOI:** 10.3389/fimmu.2019.01352

**Published:** 2019-06-25

**Authors:** Anne M. Stevens, Kathryn S. Torok, Suzanne C. Li, Sarah F. Taber, Theresa T. Lu, Francesco Zulian

**Affiliations:** ^1^Division of Rheumatology, Department of Pediatrics, University of Washington, Seattle, WA, United States; ^2^Division of Pediatric Rheumatology, Department of Pediatrics, Children's Hospital of Pittsburgh, University of Pittsburgh, Pittsburgh, PA, United States; ^3^Division of Pediatric Rheumatology, Department of Pediatrics, Hackensack University Medical Center, Hackensack Meridian School of Medicine at Seton Hall University, Hackensack, NJ, United States; ^4^Division of Pediatric Rheumatology, Department of Rheumatology, Hospital for Special Surgery, New York, NY, United States; ^5^Department of Pediatrics, Weill Cornell Medicine, New York, NY, United States; ^6^HSS Research Institute, Hospital for Special Surgery, New York, NY, United States; ^7^Department of Microbiology and Immunology, Weill Cornell Medicine, New York, NY, United States; ^8^Rheumatology Unit, Department of Woman's and Child's Health, University of Padua, Padua, Italy

**Keywords:** systemic sclerosis, Pediatric Rheumatology, genetics, disease etiology, autoimmune disease, skin, fibrosis

## Abstract

Juvenile-onset systemic sclerosis (jSSc) is a rare and severe autoimmune disease with associated life-threatening organ inflammation and evidence of fibrosis. The organ manifestations of jSSc resemble adult SSc, but with better outcomes and survival. The etiology of jSSc appears to reflect adult-onset SSc, with similar inflammatory mediators and autoantibodies, but with a significant population of children with uncharacterized anti-nuclear antibodies. The genetics of patients with jSSc differ from women with SSc, resembling instead the genes of adult males with SSc, with additional HLA genes uniquely associated with childhood-onset disease. Current treatments are aimed at inhibiting the inflammatory aspect of disease, but important mechanisms of fibrosis regulated by dermal white adipose tissue dendritic cells may provide an avenue for targeting and potentially reversing the fibrotic stage.

## Clinical Aspects

### General Demographics and Clinical Characteristics

Juvenile systemic sclerosis (jSSc) is a multisystem connective tissue disease characterized by skin induration and widespread fibrosis of internal organs. The incidence has been reported at 0.27–0.50 per million children per year in the UK and Finland (1, 2), with a prevalence of 3 per million (3–5). Less than 5% of SSc has an onset in childhood (6–8). As in adults, there are three main subtypes of jSSc: diffuse cutaneous, limited cutaneous, and overlap (6, 9–12).

The onset of jSSc is usually insidious. Isolated Raynaud phenomenon is often the presenting symptom along with positive antinuclear antibodies (ANA) and nailfold capillary changes. Months or years later, tightening of the skin, especially of fingers and face, cutaneous telangiectasias and symptoms related to internal organ involvement gradually develop [Table 1, (6, 9–13)].

**Table 1:** Clinical features of juvenile systemic sclerosis in comparison with adult-onset systemic sclerosis^*^.

	**Juvenile SSc**	**Adult-onset SSc**
	%^**^	%^**^
**Cutaneous**	**66–86**	**64–96**
Sclerodactyly	46–84	52–90
Skin induration	64–86	77–96
**Peripheral vascular**	**84**–**100**	**91**–**100**
Raynaud's phenomenon	84–100	91–100
Digital ulcers	29–50	22–41
**Respiratory**	**36**–**55**	**44**–**64**
Pulmonary arterial hypertension	2–13	14
Pulmonary fibrosis	9–26	22–36
**Cardiovascular**	**5**–**18**	**11**–**25**
Hypertension	3–8	11–17
Cardiac abnormalities	2–17	3–20
**Musculoskeletal**	**31**–**42**	**48**–**71**
Muscle weakness	20–32	15–27
Arthritis	10–35	15–17
Joint contracture	30–45	32–42
Tendon friction rubs	8–11	12–23
**Gastrointestinal**	**42–74**	**56–78**
Esophageal	24–60	65
Gastric	16–30	26
Ileum-colon	10–15	10–22
**Renal**	**3**–**5**	**2**–**13**
Proteinuria	3–5	6
Renal crisis	0–4	2–13

* *Cumulative data from references: (6, 9–13)*.

** *During the overall disease course*.

Cutaneous changes characteristically evolve in a sequence beginning with oedema, followed by induration and eventually atrophy. Nailfold capillary abnormalities are common, and include capillary dropout, tortuous dilated loops, and occasionally distorted capillary architecture (14, 15). Musculoskeletal symptoms are common in jSSc and characteristically occur at or near onset of the disease in one-third of children (6, 13).

Gastrointestinal involvement affects 42–74% of patients, and has been associated with interstitial lung disease, malnutrition, and poor quality of life (6, 11, 13, 16). Although the esophagus is often involved quite early, many patients are asymptomatic and rarely present heartburn or dysphagia. Malabsorptive diarrhea and delayed colonic transit, when present, reflect long-standing disease.

Cardiopulmonary involvement is the leading cause of morbidity and mortality in jSSc (17, 18). Cardiac inflammation and fibrosis may lead to conduction defects, arrhythmias, and impaired ventricular function. In the largest published study on jSSc, cardiac involvement was found in 8.4% of cases at onset, and in 24% during the overall course of disease (13). Cardiac involvement, especially pericarditis, is a major prognostic factor in jSSc, with a cardiac cause of death in 50% of deceased patients in a large international case series (odds ratio 41.3) (17, 18).

Pulmonary artery hypertension occurs in a minority of jSSc patients and, as in adults, may be either an isolated vascular complication or a consequence of pulmonary fibrosis (6, 13). Interstitial pulmonary fibrosis is reported in approximately one of four children (11, 13). The kidney is rarely affected (1).

### Morbidity and Survival

As compared with adults, children with jSSc have overall better outcomes, related to a lower frequency of major visceral organ involvement and lower mortality (6, 13, 17). During follow-up, interstitial lung involvement, gastroesophageal dysmotility, and renal involvement are significantly more common in adults, while arthritis and muscle inflammation are more common in children, because of the higher prevalence of the overlap form of jSSc (3, 4, 6).

Morbidity is a major issue for jSSc, with most patients having multi-organ manifestations. In a recent North American study (*n* = 64 jSSc), 38% were found to have >4 organ systems involved, likely associating with the findings that 36% of the jSSc patients were found to have impaired function (ACR functional scores >1), and 64% reported having pain in the prior week (11). Poorer QOL scores were found to be associated with gastrointestinal symptoms, arthritis and pulmonary diseases (19).

Data from the jSSc Pediatric Rheumatology European Society registry reported a 5, 10, 15, and 20 years survival of 89, 87.4, 87.4, and 82.5%, respectively—significantly better than in adult-onset disease (17). Death in jSSc is usually related to the involvement of cardiac, renal, and pulmonary systems (9, 17, 18). A small subset of jSSc patients can have a rapidly progressive disease course that leads to early death as 60% of deaths are within 5 years (13). A raised creatinine level, pericarditis, and signs of fibrosis on chest X ray at diagnosis are potential risk factors for early mortality, similar to adult-onset SSc (17). In contrast to adult disease, however, malignancy is not an additional risk factor in jSSc for mortality as it has not been associated with jSSc (9, 13, 20, 21).

### Autoantibodies and Clinical Associations

Autoimmunity plays an important role in juvenile and adult SSc, with a high prevalence of positive ANA and scleroderma-related autoantibodies, which can further assist clinical characterization and organ risk assessment as an adjunct to skin thickening distribution assignment of limited or diffuse cutaneous (6, 11, 13, 22–24). For example, Anti-topoisomerase antibodies (ATA; Scl-70) would be expected in a patient with diffuse cutaneous SSc, and would be worrisome for rapid skin progression and development of interstitial lung disease (ILD), prompting more aggressive pulmonary monitoring (6, 13, 23).

ANA positivity is reported in 78–97% of patients in the jSSc cohorts (6, 9–13), with scleroderma-associated antibodies reflecting the majority of extractable nuclear antigens causing ANA reactivity, such as ATA (Scl-70), centromere, and U1-RNP as reported in adult SSc, but in divergent frequencies, mirroring the slightly different clinical phenotype in children with SSc (Table 2). Another observation is that a significant proportion of jSSc patients, up to 23%, have a positive ANA without a specific extractable nuclear antigen identified, which is rarely observed in adults (6, 11, 13).

**Table 2:** Comparison between pediatric and adult-onset systemic sclerosis (SSc) subtype and autoantibody distribution.

	**Pediatric-Onset SSc**	**Adult-Onset SSc**
**Clinical Subsets (%)**		
Diffuse cutaneous SSc	30–65	35–45
Limited cutaneous SSc	30–50	40–55
**Overlap**	**10**–39^*^	**9**–**18**
**Antibody Positivity (%)**		
Antinuclear antibody	78–97	90–99
Anti-topoisomerase (ATA, Scl-70)	20–46	20–40
**Anti-centromere (ACA)**	**2**–**15**^†^	**20**–**30**
**Anti-U1 ribonucleoprotein (U1-RNP)**	**15**–20^*^	**5**
**Anti-polymyositis-scleroderma (PM-Scl)**	15^*^	**5**
**RNA polymerase III (POL3)**	**2**–**4**^†^	**10**–**30**

* *Significantly higher in pediatric onset SSc compared to adult-onset SSc group*.

† *Significantly lower in pediatric onset SSc compared to adult-onset SSc group*.

*Boldface text highlights a difference between pediatric and adult SSc*.

*Pediatric Onset data from: juvenile SSc cohorts (6, 9–13)*.

Table 2 shows the general distribution of SSc clinical subtypes and the SSc-associated auto-antibodies in pediatric-onset compared to adult SSc. The diffuse cutaneous SSc subtype and associated ATA frequencies are similar between pediatric and adult SSc. A higher proportion of overlap patients and associated antibodies U1-RNP and PM-Scl was observed across several jSSc cohorts (6, 9–12) and accounts for the relatively common disease presentation in children of the combination of arthritis, myositis, Raynaud phenomenon and digital ulcers (6). The jSSc patients with overlap syndrome and associated antibodies (U1-RNP, Pm-Scl, U3-RNP) that have myositis may be of the most concern, as myopathy of the peripheral skeletal muscle is likely related to cardiac skeletal myopathy, and these patients have been demonstrated to have more conduction defects and other cardiac manifestations, a major contributor to mortality (6, 12, 13, 18).

Another main difference from adults with SSc is the lack of anti-centromere antibody–positive patients with jSSc (*leq*5% in most cohorts) (6, 9–13, 25), especially with pre-pubertal patients (<10 years at onset) (10). This is true despite ~30% to 50% of patients with jSSc being clinically classified as limited cutaneous SSc (Table 2). The relatively low rate of centromere antibodies found in jSSc does not seem to correlate with pulmonary arterial hypertension (PAH), as PAH is found with a similar frequency in juvenile and adult SSc (Table 1) (6, 9–13, 23). Specifically, in the Scalapino jSSc cohort manuscript (*n* = 111, 4 with intrinsic PAH) it was noted there was no association of ACA positivity with manifestation of PAH (6).

There are several other SSc-related autoantibodies that associate with different organ manifestations in adult SSc; however, they are much less common in childhood onset SSc. One of these is RNA polymerase III (POL3) antibody, which relates to severe renal disease in the form of scleroderma renal crisis in adult SSc. POL3 is rarely observed in jSSc (<5% in all cohorts; 4 or fewer patients per cohort), but can be found in up to 30% of adult onset SSc patients and reflects the clinically significant higher proportion of renal disease in adult SSc (6, 23). POL3 antibodies have also been associated with malignancy in SSc, a clinical outcome not reported in jSSc (21). Some of this data in jSSc is limited by the incomplete serological testing of scleroderma-associated antibodies in children. A more comprehensive evaluation for less common autoantibodies, such as POL3 and Th/To, to complete the full scleroderma-associated auto-antibody profile in jSSc cohorts may allow for better disease characterization and allow for more significant clinical associations.

## Genetic Basis of Juvenile Systemic Sclerosis

### Evidence for a Genetic Contribution to jSSc

Although the etiology of SSc is undoubtedly multifactorial, association studies have revealed immunoregulatory genes that most likely contribute to the pathogenesis of disease. The genetic risk factors appear to be lower for adult-onset SSc than for other autoimmune diseases [reviewed recently in (26)]. Only one study has examined history of autoimmunity in family members of SSc patients, and found a rate of 11% for a first or second degree relative of jSSc patients, suggesting that the disease is not monogenic (13).

The concordance rate for adult-onset SSc in twins is low (5.6% for dizygotic (DZ) twins and 4.2% for monozygotic (MZ) twins) (27). However, the genetic predisposition for loss of immune self-tolerance is high in families with SSc, with greatly increased concordance for ANA in MZ twins (90%) compared with DZ twins (40%) (27). Some of the discordance in disease penetrance may be attributed to differential methylation of X-encoded genes (28). A genetic contribution has also been suggested by reports of familial clustering in three cohorts in which first degree relatives of patients with SSc carried a 10–16-fold relative risk for disease and siblings a 10–27-fold risk (29).

In families with more than one case of SSc, affected individuals shared cutaneous subsets of disease severity and SSc-associated autoantibodies, with similar ages of onset (30). Finally, gene expression studies in cultured dermal fibroblasts suggest a heritable profibrotic program. In this study, healthy adult-onset MZ twins shared fibroblast gene expression patterns with their twins with SSc, whereas gene expression in unaffected adult-onset DZ twins resembled that of healthy controls (31). Moreover, serum from SSc patients or MZ twins could induce a SSc gene expression pattern in fibroblasts from healthy controls (31).

### Peculiar Genetic Aspects of Juvenile SSc

Although numerous studies have investigated HLA associations in adult SSc, age of onset has not generally been considered. In pediatric studies, some HLA alleles found associated with adult SSc have also been associated with jSSc (32). An initial study in the UK examined HLA alleles in 27 patients with jSSc-dermatomyositis overlap, demonstrated an increase of DQA1^*^05 and DRB1^*^03 and a trend toward decreased DRB1^*^15. This finding was confirmed in a larger study of 76 Caucasian patients with a mean onset of SSc onset at 10 years of age in the US (25). Children with SSc did not have an increased frequency of DRB1^*^01, which has been associated with adult limited cutaneous SSc and ACA or HLA-DRB1^*^11 alleles, the group most consistently described in association with SSc in Caucasian adults.

One novel HLA association was discovered in jSSc patients: DRB1^*^10. DRB1^*^10 appeared to be a risk factor specific for jSSc, present in 10.5% of jSSc compared to 1.5% of controls, similar to one report of adult Han Chinese, but not otherwise described in adult SSc (33).

Protective HLA alleles in adult-onset SSc (DRB1^*^07, DQB1^*^02:02) were not found to be protective for jSSc. Instead, DQB1^*^06 was protective for jSSc (25).

### Age and Sex Matter

The frequency of the adult limited cutaneous SSc-associated allele DRB1^*^01 differed in children with age of disease onset and autoantibody status (25). DRB1^*^01 was less prevalent in patients who were <6 years of age compared to patients 11–16 years at disease onset. DRB1^*^01 was under-represented among anti-topoisomerase 1 (ATA) -positive jSSc patients, consistent with a similar observation reported in adult SSc (34). In this study, just as the ATA-associated alleles decreased with age, the prevalence of ATA significantly decreased with increasing age (25).

Thus, children with SSc genetically resemble adult males with SSc, rather than the more prevalent women with SSc, with an increase frequency of DQA1^*^05 but not DRB1^*^11. This phenomenon could be explained by pregnancy immunopathology. It has been hypothesized that fetal microchimerism could trigger or perpetuate a graft-vs. -host type chronic inflammatory response (35). Women with SSc more often have been pregnant, and carry high levels of fetal microchimerism, whereas males and children do not. About 1 in 10 children with SSc carry an HLA allele not found in adults with SSc (DRB1^*^10), suggesting a unique childhood antigen trigger.

## Pathology of Systemic Sclerosis

### Histology of the Skin in SSc

The histology of the skin reflects the main three pathways affected in SSc, endothelial/vascular, immune/inflammatory, and fibrosis. The epidermis is relatively spared, though may be thinned, and rete ridges reduced. The dermis is thickened from the fibrosis and the connective tissue appears homogenous from collagen deposition. There is a loss of adnexal structures such as the pilosebaceous and eccrine glands (36). There are also vascular changes characterized by capillary rarefaction in the papillary dermis, thickened arteriolar walls and intimal thickening (37, 38). There can be inflammatory infiltrates of lymphocytes and macrophages, similar to that found in localized scleroderma (37), though may limited to early disease in SSc.

### The Role of the Adipocytes

The dermal alterations that are most often discussed are the features that are apparent on a full thickness skin biopsy. However, intrinsic to the dermis but sitting mainly under the connective tissue-rich portions of the dermis and immediately adjacent to subcutaneous adipose tissue in humans is the dermal white adipose tissue (DWAT) (39). Fleishmajer et al. performed excisional biopsies in SSc patients, sampling skin down to the fascia, and thereby were able to characterize the DWAT (36). They reported that DWAT was replaced by connective tissue comprised of immature disorganized collagen fibrils and massive ground substance. Interestingly, this loss of DWAT is also observed in multiple murine models of scleroderma skin fibrosis (40–42), and reflects at least in part transdifferentiation of adipocytes into myofibroblasts (40, 41). Furthermore, this DWAT loss can further compromise skin function, as adipocytes can express anti-fibrotic molecules such as adiponectin and are involved in hair regeneration. DWAT is a niche for regenerative and reparative adipose-derived stromal cells (ADSCs) that participate in wound healing (41, 43, 44).

**Figure 1:**
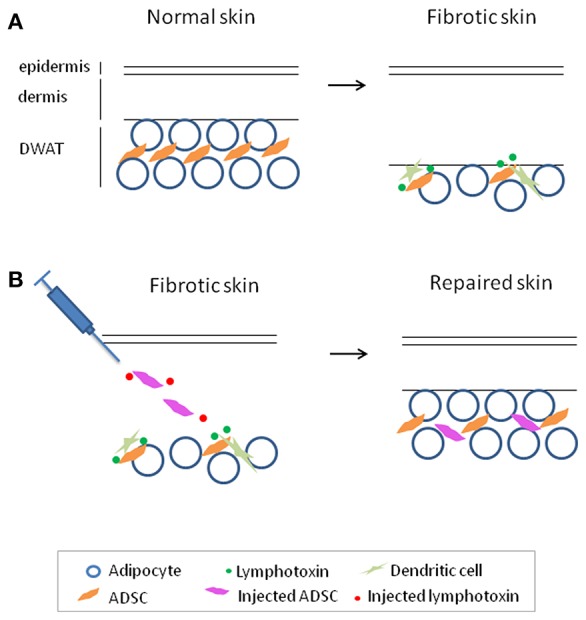
Adipose-derived stromal cells (ADSC) maintenance by dendritic cell-derived lymphotoxin in fibrotic skin and enhancing ADSC therapy with lymphotoxin stimulation to treat fibrosis. **(A)** Reparative and regenerative ADSCs are lost with skin fibrosis and are maintained in fibrotic skin by dendritic cell-derived lymphotoxin. **(B)** Adding lymphotoxin stimulation to therapeutic ADSC injection enhances survival and effectiveness of the injected ADSCs in skin repair in a model of skin fibrosis. This model may be helpful to investigate its potential application for the treatment of fibrosis.

Upon loss of DWAT in murine skin fibrosis models, there is an 80% loss of ADSCs, likely due to cell death, which could potentially contribute to the inability to reverse and heal the fibrosis (42). Interestingly, dendritic cells helped to maintain the survival of the remaining ADSCs in fibrotic skin, and stimulating with the dendritic cell-derived signal lymphotoxin can enhance the survival and therapeutic effectiveness of intradermally injected ADSCs in a murine skin fibrosis model. ADSCs or a related type of mesenchymal stromal cells (MSCs) given either locally or systemically are being tested in human SSc (45); if clinical trials show efficacy of these approaches, it may be helpful to investigate whether adding lymphotoxin beta receptor stimulation can enhance these approaches (Figure 1).

## Cytokine and Cellular Signatures

There have been multiple studies in adult SSc evaluating the cellular and cytokine profiles of tissue and peripheral blood, with only a few studies in jSSc owing to the rarity of disease in children. Skin lesions in jSSc show lymphocytes as the predominant cell type within the dermal and subcutaneous infiltrate (46, 47).

The lymphocytes have been further characterized, with T lymphocytes (CD4 and CD8) as the predominant cell type in the dermis of SSc patients (48, 49). T cell activation and associated cytokine release are thought to play a pivotal role in SSc pathogenesis through stimulating fibroblasts to promote fibrosis (50–52). CD4+ T-helper (T_H_) cell lineage and its effector cytokines have been identified in biopsies of skin lesions (48, 53, 54), peripheral circulation (adult and pediatric) (55–58) and culture in supernatants of peripheral blood mononuclear cells (PBMC) of SSc patients (59).

T_H_ cells consist of three main effector cell types including T_H_1, T_H_2, and T_H_17; each are differentiation states characterized by the predominant cytokines they produce, namely interferon-γ (IFN-γ), interleukin-4 (IL-4)/IL-13, and IL-17, respectively. Many autoimmune diseases, including scleroderma, are thought to be propagated by an imbalance of T_H_ cell subsets and their associated cytokines (48, 53–57, 59–61).

There is an extensive amount of literature (mainly adult studies) supporting a T_H_2 cellular and associated cytokine predominance in SSc, with peripheral blood and tissue derived IL-4 and IL-13, and more recently identified T_H_2 associated cytokines, IL-33, IL-34, and IL-35 (62, 63), correlating with the degree of skin and lung fibrosis and disease burden (50, 51, 57, 64–67). Both IL-4 and IL-13 are effector cytokines of the T_H_2 lineage characterized as pro-fibrotic and anti-inflammatory due to their respective actions as initiators of extracellular matrix production and inhibitors of T_H_1 function (68).

Therefore, it is suspected that the T_H_2 cytokine signature in SSc supports the fibrotic component of the disease, with peripheral cytokines serving as prognostic markers in SSc patients (50, 51). A recent study analyzing 14 pediatric SSc subjects compared to 24 healthy pediatric controls found a significantly elevated proportion of circulating CD4+IL-4+ (T_H_2) cells in pediatric SSc, and notably 10 of the 14 subjects had later stage disease (>2 years from onset) (58). Conversely, although higher than pediatric LS, the T_H_17 (CD4+IL-17+) profile of these pediatric SSc patients was significantly lower than pediatric healthy controls, perhaps reflecting their later stage of disease (58).

Cytokines associated with a T_H_1 profile (IL-1, TNF-α, and IFN-γ) have been identified as elevated in the peripheral blood of adult SSc patients compared to healthy controls, and their levels decrease overtime signifying their elevation during the earlier, active phase of the disease (69, 70). Specifically, the IFN-γ associated chemokines, IFN-inducible protein 10 (IP-10/CXCL10) and monocyte chemoattractant protein 1 (MCP-1/CCL2), have been demonstrated in more early, inflammatory adult SSc (71) as well as preliminary data in jSSc (72). The sera levels of jSSc are even more elevated than adult SSc and reflect those of juvenile LS (73), which in general, is considered the more inflammatory scleroderma subset. The “hallmark” effector cytokine of T_H_17 cells, IL-17a, has been demonstrated in significant amounts in the skin, lungs, and sera of adult SSc patients during the early, more active, stages of the disease (55). These findings suggest that T_H_17 and T_H_1 cells may contribute to cellular inflammation in SSc via production of associated pro-inflammatory cytokines (51) in the earlier stage of disease, as hypothesized in the pediatric SSc findings (58, 72).

It is thought that these T cell–associated chemokines and cytokines then stimulate fibroblasts and endothelial cells to produce TGF-β and connective tissue growth factor (CTGF) to stimulate tissue fibrosis (via increased collagen production) and endothelial cell damage (via influence of adhesion molecules ICAM-1 (intercellular adhesion molecule-1) and VCAM-1 (vascular cell adhesion molecule-1), with elevation of these adhesion molecules demonstrated in the peripheral blood of SSc patients (74–78).

The counterbalance to the inflammatory T_H_ cell phenotype, the regulatory T cell (Treg) immunophenotype, has been recently reported by Torok and Reiff in jSSc patients (58, 79), and collectively demonstrate decrease in functional Tregs compared to healthy controls, as well as association to more severe clinical phenotype and longer disease duration. This could, in part, be due to the differentiation of Tregs in the blood and skin to proinflammatory (T_H_17) and fibrotic (T_H_2) cells, therefore decreasing their frequency and effectiveness, as reported in adult SSc (80, 81).

Although the focus has been more on lymphocytes, innate cells such as macrophages and plasmacytoid dendritic cells are becoming increasingly recognized as potential contributors to systemic sclerosis, especially during early disease (42, 82–85). In addition to directly stimulating fibroblasts, innate cells can contribute to activating endothelial cells, which then further recruits circulating cells into the tissue, fueling the inflammation and further injury of the endothelium (86–88). While most patient data is gathered from adult SSc patients, a recent gene expression and expression quantitative trait loci analysis of monocyte-derived macrophages generated from pediatric and adult SSc patients suggested that changes in macrophage gene expression is an important contributor to disease and that upregulation of GSMDA, a regulator of pyroptosis in macrophages but not other cell types, contributes to disease risk (89). Further delineation of innate cell phenotype and function in pediatric SSc awaits.

**Figure 2:**
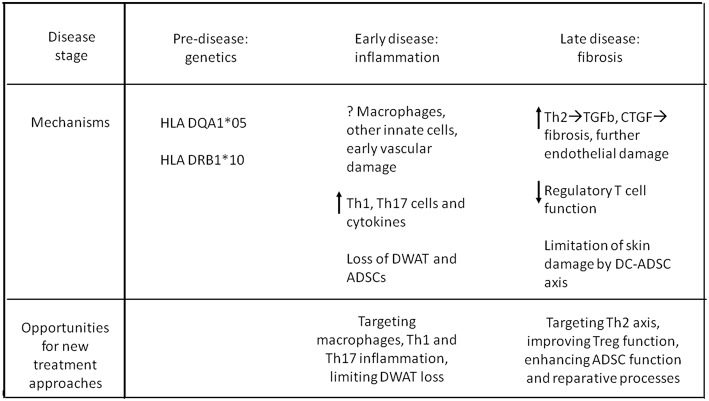
Schematic representation of proposed disease pathogenesis of pediatric SSc.

Figure 2 summarizes, in a schematic way, the main immunologic/genetic pathways involved in the pathogenesis of the disease, in its different stages.

## Future Directions For Research

In summary, early-onset SSc seems to be a distinct entity, whereas genetically-programmed manifestations in older children resemble adult disease. Disease manifestations and histopathology of jSSc resemble adult SSc, albeit with less organ involvement and superior outcomes.

Although genome-wide association studies cannot be performed on a disease as rare as jSSc, a genetic basis is likely in early-onset disease, and HLA class II associations with jSSc resemble adult males rather than adult females with SSc.

A pediatric-specific association of DRB1^*^10 suggests a unique trigger leading to younger onset disease. Younger children with SSc tended to carry genes associated with ATA, diffuse cutaneous SSc type, and not DRB1^*^01. More extensive genetic studies in families of children with early-onset (<6 years old) disease, with special focus on those with rapidly progressive organ fibrosis (90) are needed.

Novel therapeutic approaches could target specific HLA molecules, inflammatory chemokine, and cytokine genes over-expressed in jSSc. Recent reports of promising results of hematopoietic stem cell transplantation in adult-onset SSc encourage optimism for this therapeutic approach in children, who may have less organ damage when disease is recognized (91) Anti-fibrotic cellular therapies with adipose-derived stromal cells are being investigated, and recent findings in the laboratory suggest the potential power of stromal cell survival factors to enhance cellular therapy of the future.

## Author Contributions

All authors listed have made a substantial, direct and intellectual contribution to the work, and approved it for publication.

### Conflict of Interest Statement

The authors declare that the research was conducted in the absence of any commercial or financial relationships that could be construed as a potential conflict of interest.
